# Antibacterial Properties of Canine Platelet-Rich Plasma and Other Non-Transfusional Hemo-Components: An *in vitro* Study

**DOI:** 10.3389/fvets.2021.746809

**Published:** 2021-10-04

**Authors:** Anna-Rita Attili, Cristina Iacoucci, Evelina Serri, Vincenzo Cuteri, Andrea Cantalamessa, Martina Linardi, Claudia Rifici, Giuseppe Mazzullo, Giacomo Rossi, Livio Galosi, Adolfo Maria Tambella

**Affiliations:** ^1^Laboratory of Medical Microbiology and Infectious Diseases, School of Biosciences and Veterinary Medicine, University of Camerino, Matelica, Italy; ^2^Veterinary Teaching Hospital, School of Biosciences and Veterinary Medicine, University of Camerino, Matelica, Italy; ^3^Pathology Laboratory, Department of Veterinary Sciences, University of Messina, Messina, Italy; ^4^Pathology Laboratory, School of Biosciences and Veterinary Medicine, University of Camerino, Matelica, Italy

**Keywords:** platelet-rich plasma, platelet-poor plasma, platelet lysate, platelet concentrates, leucocytes, antibacterial effect, dogs

## Abstract

This *in vitro* study was carried out to evaluate the potential antibacterial properties of canine non-transfusional hemo-components. Therapeutic formulations commonly used for regenerative medicine purposes (platelet-rich plasma, platelet gel, platelet lysate, fibrin glue), considering both leukocyte-rich and leukocyte-poor formulations, but also platelet-poor plasma and activating substances (thrombin, calcium gluconate), were tested to detect elements with potential antimicrobial properties. The antibacterial effect was tested on different bacterial strains (*Staphylococcus aureus* subspecies *aureus, Staphylococcus cohnii* subspecies *cohnii, Escherichia coli, Pseudomonas aeruginosa*, and *Klebsiella pneumoniae* subspecies *pneumoniae*) isolated from canine wounds and classified as susceptible, multidrug-, extensively, and pandrug-resistant bacteria toward a known panel of human and veterinary antibiotics. The evaluation was carried out by agar gel diffusion method (Kirby–Bauer) and micro-inhibition in broth using microplates and spectrophotometer reading. The study findings confirmed the hypothesized antibacterial properties of canine non-transfusional hemo-components. A more effective bacteriostatic effect was found against Gram-negative bacteria, drug-resistant too. The presence of leukocytes or platelets does not appear to be essential for the antibacterial effect. Further studies should be conducted to evaluate the exact mechanism of action of the antimicrobial activity. However, non-transfusional hemo-components could be a useful natural aid in controlling bacterial infections in dogs.

## Introduction

Antimicrobial resistance (AMR) is a multifaceted topic at the interface of human, animal, and plant health, food hygiene, and environmental sciences, resulting to be a major global public health issue of the 21st century (1, 2).

To counter this increasing phenomenon, both in human and in veterinary medicine, more and more scientists are looking for molecules that can assist or replace the action of antibiotics. The topical use of non-transfusional hemo-components or autologous platelet concentrates (PCs), including platelet-rich plasma (PRP), platelet gel (PG), platelet lysate (PL), and fibrin glue (FG), has gained great popularity during the past 20 years; thus, they have been used, mainly for their regenerative properties, in a variety of human medical fields such as orthopedics (3–7), wound healing (8–12), ophthalmology (13–15), and dentistry (16–20). Despite a recent growing interest, fewer *in vivo* and *in vitro* studies in dogs have been published (21–26).

While the potential regenerative effect of PCs has been extensively studied, fewer studies have investigated the possible relationship with bacterial growth. Some authors have empirically supposed that PCs could promote infections, associating them with microbiological culture media and acting as a substrate for the growth of bacteria (27). More recently, on the contrary to this theory, some evidence would attribute to PCs an inhibitory effect on bacterial growth, although the results cannot be considered definitive yet (27–31). The antibacterial properties of PCs are supported by some *in vitro* studies conducted mostly in humans (28, 30, 32–50), but also in horses (31, 51, 52) and rabbits (53). Systematic reviews of current literature considering *in vitro*, preclinical, and clinical studies have shown that PCs may have antibacterial properties (54–56).

The antibacterial effect of PCs has also been recently highlighted in *in vivo* studies on surgical wounds (57), sternotomy wounds (58, 59), osteomyelitis (60–62), spinal infections (63), and bacterial cystitis (64), while another *in vivo* study showed controversial results (65). Since infection control is a prerequisite for the wound healing process and for avoiding the chronicization event, recently, in a small controlled clinical trial performed in dogs, MRSA-infected skin wounds treated with PRP showed an accelerated healing process with rapid re-epithelialization and granulation tissue formation, reduction of inflammation, and decrease of bacterial loads (66).

Until now, the components of the PCs that control antimicrobial activity have not been fully understood, so the mechanisms of PC interaction with bacteria need further investigation.

In order to detect any elements potentially endowed with antimicrobial properties, we explored the antimicrobial activity of PCs (PRP, PG, PL, FG) against susceptible (S), multidrug-resistant (MDR) extensively drug-resistant (XDR), and pandrug-resistant (PDR) Gram-positive and Gram-negative microorganisms isolated from canine wounds. In this set of *in vitro* investigations, leukocyte-rich (L-PRP, L-PG) and leukocyte-poor formulations (P-PRP, P-PG), but also platelet-poor plasma (L-PPP, P-PPP) and intermediate elements of the PC production process (thrombin, calcium gluconate), were evaluated.

## Materials and Methods

### Animals and Blood Collection

The study was performed complying with the Animal Welfare Body of the University of Camerino (protocol number E81AC.10/A) according to the National Legislative Decree n. 26/2014, implementation of EU Directive 2010/63/EU. Blood was collected from five owned dogs at the Veterinary Teaching Hospital, School of Biosciences and Veterinary Medicine, University of Camerino. The owner's informed consent was obtained for participation in the study. All animals were between 1 and 10 years of age, had a minimum body weight of 15 kg, and belonged to different dog breeds (three mixed breed, one Labrador retriever, one English setter). They had no noteworthy medical problems or medical history, except for degenerative joint disease, and did not take any medications. An articular infiltration of autologous PRP was practiced in each dog for the management of degenerative joint disease; an aliquot of whole blood in excess of the amount needed for PRP preparation was used for the *in vitro* investigations of the present study. Each dog, upon admission, had a complete physical examination, complete blood count, and serum biochemical profile. No animals had platelet disorders or took anticoagulant and antibiotic drugs.

### Preparation of Hemo-Components

In each dog, fresh autologous whole blood (50 ml) was collected from the jugular vein using a 60-ml syringe containing anticoagulant citrate dextrose solution A (ACD-A: SALF SpA, Cenate Sotto, Bergamo, Italy) in a ratio of 1:9. Blood collection and preparation procedures were performed as described by Tambella and collaborators (22) in aseptic conditions through a laminar flow cabin (Bicasa, Bernareggio, MB, Italy) and following the Good Laboratory Practices. The hemo-components were obtained from a pool of fresh canine whole blood. Prior to preparation of each hemo-component, an aliquot was used to determine the blood cell count.

#### Autologous Thrombin

The thrombin-rich solution was obtained from autologous whole blood which was centrifuged (Rotina 46R, Hettich, Milan, Italy) at 650 *g* for 10 min. The plasma supernatant fraction was mixed with 10% calcium gluconate (B. Braun, Melsungen, Germany) in a ratio of 5:1 and incubated (cooled incubator IL 23R, VWR Incu-Line, Milan, Italy) at 37°C for 30 min. The resulting clot was crushed, and the final supernatant, the thrombin-rich solution, was collected for PRP activation.

#### Leukocyte-Rich -PPP, -PRP, and -PG

As described by Tambella et al. (22), leukocyte-rich platelet-poor plasma (L-PPP), leukocyte PRP (L-PRP), and leukocyte PG (L-PG) were obtained by a double-spin technique. Whole blood was centrifuged (Rotina 46R, Hettich, Milan, Italy) at 180 *g* for 20 min. To stratify the concentrated platelet pellet in the bottom layer, and platelet poor plasma (L-PPP) in the supernatant layer, the plasma with the buffy coat layer was centrifuged at 650 *g* for 15 min. A half amount of L-PPP was picked up and used for the *in vitro* study. By resuspending the PCs in the residual part of PPP, the L-PRP was obtained. A half amount of L-PRP was used for the *in vitro* study; to obtain L-PG, the residual L-PRP was transferred in sterile glass Petri dishes and mixed with the thrombin-rich solution and the calcium gluconate in a volumetric ratio of 8:1:0.5. The L-PG production was achieved at room temperature in 5 min.

#### Leukocyte-Poor (Pure) -PPP, -PRP, and -PG

Pure platelet-poor plasma (P-PPP), pure platelet-rich plasma (P-PRP), and pure platelet gel (P-PG) were obtained by a double-spin technique. In order to reduce the leukocyte concentration as much as possible, the first centrifugation of whole blood was performed at 500 *g* for 20 min using tubes (VF108SAS Venosafe, Terumo Europe NV, Leuven, Belgium) containing a gel enabling the separation of the different blood components. The plasma fraction was then centrifuged at 2,000 *g* for 10 min to achieve separation of P-PPP (the supernatant half-fraction) from P-PRP (the residual half-fraction). Similarly, P-PG was obtained by mixing P-PRP with the autologous thrombin-rich solution and calcium gluconate.

#### FG

Fibrinogen concentrate, the precursor of FG, was obtained following the method proposed by Tarantino et al. (67). This method is characterized by physical separation (centrifugation) and the presence of a special membrane, capable of concentrating fibrinogen. After centrifugation of whole blood (2,000 *g* for 10 min), the plasma fraction obtained without buffy coat was transferred in special tubes (Amicon Ultra 15, Merck Millipore Ltd, Tullagreen, Carrigtwohill, Co Cork, Ireland) with a 100,000 MWCO (molecular weight cutoff) porosity filter. Then, tubes were centrifuged at 3,600 *g* for 45 min and the fibrinogen concentrate above the filter was obtained.

#### PL

PL was produced as reported in previous studies with some modifications (68, 69). Using a two-step centrifugation protocol (180 *g* for 20 minutes and 1,500 *g* for 10 minutes), packed PC was produced from whole blood with ACD-A. To remove platelet membranes and other cellular debris and obtain the PL, three repeated cycles of freezing (at −20°C) and thawing (at room temperature) followed by centrifugation at 2,500 *g* for 20 min were undergone obtaining the PL as supernatant.

### Evaluation of Antibacterial Activity

#### Bacterial Strains and Their Antibiotic Susceptibility Evaluation

To compare the antibacterial effect of each hemo-component, strains isolated from canine wounds were selected from the bacterial collection of the Laboratory of Microbiology and Infectious Diseases, School of Bioscience and Veterinary Medicine, University of Camerino, based on their ability to cause also infections in the surgical setting.

Three different frozen strains of *Staphylococcus aureus* subspecies *aureus*, one *Staphylococcus cohnii* subspecies *cohnii*, three *Klebsiella pneumoniae* subspecies *pneumoniae*, two *Pseudomonas aeruginosa*, and three *Escherichia coli* strains were thawed and reconstituted. In addition, *Staphylococcus aureus* subspecies *aureus* ATCC® 43300^™^ strain was used as control. After pre-enrichment in Tryptic Soy Broth (TSB) (Liofilchem, Roseto degli Abruzzi, Italy) and incubation at 37°C for 6 h in aerobic condition, a loop was shown on Columbia Blood Agar (Liofilchem, Italy), Mannitol Salt Agar (Liofilchem, Italy), MacConkey Agar (Liofilchem, Italy), and Pseudomonas Cetrimide Agar (Liofilchem, Italy) and incubated aerobically at 37°C for 24 h. Colonies were identified and confirmed by MALDI-TOF MS (Bruker Daltonics, Hamburg, Germany).

In order to determine their antibiotic susceptibility profile and classify the strains as susceptible, MDR, XDR, and PDR (70), antimicrobial susceptibility testing toward 16 commercial human and veterinary antibiotics, belonging to eight different classes, was performed according to the Clinical and Laboratory Standards Institute and EUCAST guidelines (71, 72).The standard disk diffusion method (Kirby–Bauer test) was used to test amoxicillin and clavulanic acid (AUG 30 μg), penicillin (P 1 UI), oxacillin (OX 1 μg), cefadroxil (CDX 30 μg), cefoxitin (CFX 30 μg), cefquinome (CEQ 30 μg), enrofloxacin (ENR 5 μg), gentamicin (CN 10 μg), sulfamethoxazole and trimethoprim (SXT 25 μg), tetracycline (TE 30 μg), amikacin (AK 30 μg), streptomycin (S 300 μg), metronidazole (M 50 μg), and polymyxin B (PB 300 μg). The E-test method was used for *Staphylococcus* strains to determine the MICs against vancomycin (VA), and teicoplanin (TEC), as described by the manufacturer (MIC Test Strip, Liofilchem, Italy).

#### Antibacterial *in vitro* Evaluation of Hemo-Components

To evaluate the antibacterial effect of different hemo-components and activating agents (PL, FG, thrombin, L-PPP, P-PPP, L-PRP with calcium gluconate, P-PRP with calcium gluconate, L-PRP with thrombin, P-PRP with thrombin, L-PG 35 μl, P-PG 35 μl, L-PG 500 μl, and P-PG 500 μl), the Kirby–Bauer disk diffusion method and the broth inhibition by microtiter method were performed.

##### Kirby–Bauer Disk Diffusion Method

Following the CLSI (2018) recommendations (71), each microorganism was suspended in sterile saline solution (Thermo Fisher, Milan, Italy) to obtain an optical density equal to 0.5 McFarland (1 × 10^8^ CFU/ml), verified at 550 nm by a spectrophotometer (Jenway, Genova Nano, Bibby Scientific, Staffordshire, UK). A sterile cotton swab was dipped into the inoculum suspension and the excess fluid removed by turning the swab against the inside of the tube to avoid over-inoculation of plates. The inoculum was spread evenly over the entire surface of the Mueller Hinton II agar plate (MH; Liofilchem, Italy) by swabbing in three directions. Each hemo-component was placed by a micropipette (PIPETMAN L P20L 2–20 μl, Gilson, Milan, Italy) on the surface of the media, in contact with the microorganisms to reduce as much as possible the variables that could influence their actions against bacteria. Specifically, four different Petri dishes were used to test hemo-components with and without leukocytes against each microorganism.

The amount of each liquid hemo-component was 8 μl, except for PG, tested at 35 and 500 μk, in the pre-jellification phase. Positive controls were performed inoculating the microbial suspension (McFarland 0.5) on MH (Liofilchem, Italy) alone, whereas the same media inoculated with saline solution or hemo-components, with and without leukocytes only, represented the negative controls. The assay was conducted in duplicate and repeated twice. All plates were incubated at 37°C aerobically and evaluated after 4, 8, and 24 h. For each strain, areas (mm) with bacterial inhibition or areas with few bacterial colonies were compared to the respective positive control.

##### Broth Inhibition by Microtiter Method

The antibacterial activity was determined using the broth inhibition by microtiter method. Bacterial strains have been grown in Tryptic Soy Broth (Liofilchem, Italy) at 37°C for 6 h, aerobically. The final inoculum concentration was adjusted using a spectrophotometer (Jenway, Genova Nano, Bibby Scientific, Staffordshire, UK) to 1 × 10^4^ CFU/ml (OD_540nm_), and 20 μl of each bacterial suspension was inoculated into a 96-well microtiter plate (Sero-Wel®, Bibby Sterilin Ltd., UK) containing 10 μl of each hemo-component (PL, FG, L-PRP, P-PRP, L-PRP plus thrombin, P-PRP plus thrombin, L-PRP plus calcium gluconate, P-PRP plus calcium gluconate, L-PPP, P-PPP) and 170 μl of sterile TSB. Quantities of 10, 20, 40, and 180 μl were used for pre-jellification L-PG and P-PG to test the antimicrobial activity toward 20 μl of each bacterial suspension. Each well was tested with the respective negative control represented by the same quantities of the hemo-components only. Positive controls with 20 μl of each bacterial suspension in 170 μl of sterile TSB were considered. As a blank negative control, an amount of 170 μl of sterile TSB was placed in the first and last wells of each plate. In addition, wells containing only each hemo-component were used as negative controls. No 0.01% acetic acid was added to prevent peptide aggregation and release of platelet contents. The microtiter plates were incubated at 37°C aerobically under slow and continuous agitation (300 g/minute) (Asal 715, Milano, Italy) and evaluated after 4, 18, and 24 h of incubation. A visual assessment (48) was conducted after 4 h, while at 18 and 24 h the observations were read using a spectrophotometer (Multiskan Ascent, Thermo Scientific, Waltham, MA, USA), at a wavelength of 540 nm. The test was repeated twice.

### Statistical Analysis

All experiments were performed in duplicate. Statistical differences between quantitative variables (mean OD_540nm_) were evaluated with Student's *t*-test. To determine the antimicrobial activity, the mean optical density (OD_540nm_) value recorded for each microorganism and the hemo-component, less the OD_540nm_ value recorded for each hemo-component, was compared to the respective positive controls recorded at the same time. Mean OD values for the different bacteria (susceptible or drug-resistant Gram-positive and Gram-negative strains) and recorded at different times (18–24 h) were analyzed with and without the leukocyte component.

Finally, the mean OD values of bacterial concentrations in the presence of hemo-components with leukocytes were compared with the mean OD values observed in the presence of hemo-components without leukocytes. STATA version 13.0 (STATA Corporation, College Station, TX, USA) was used to conduct the statistical analysis. A *p* value less than 0.05 was considered statistically significant.

## Results

### Preparation of Hemo-Components and Whole Blood and Hemo-Component Cell Counts

No technical problems occurred during the preparation of non-transfusional hemo-components. Each hemo-component was easily produced using common laboratory tools and was ready for use in this *in vitro* study. Cell concentrations were appropriate for the specific characteristics requested for each hemo-component.

In L-PRP and L-PG, platelet concentrations increased by 4.3-fold and leukocyte concentrations increased 2.1-fold compared with whole blood (WB) baseline values.

In L-PPP, platelet count decreased approximately 142-fold compared with L-PRP and leukocytes decreased approximately 45-fold.

As expected, the pure formulations (P-PRP, P-PG, and P-PPP) were leukocyte-depleted. Platelet concentrations in P-PRP and P-PG increased 2.2-fold from WB.

Both platelet and leukocyte concentrations were very low in P-PPP and FG.

There were no detectable blood cells in PL.

The mean number of platelets and white blood cells obtained by blood count analysis from the WB pool and the different hemo-components are reported in Table 1.

**Table 1:** Leukocyte and platelet concentrations obtained from whole blood (pool of 250 ml from five dogs) and each hemo-component.

**Cell count evaluation**								
	**WB**	**L-PRP/L-PG**	**L-PPP**	**P-PRP/P-PG**	**P-PPP**	**FG**	**PL**	**T**
**WBC**	8.71	18.29	0.40	0.18	0.10	0.07	nd	nd
**PLT**	265	1139	8	583	1	3	nd	nd

*WBC = number of leukocytes ×10^3^/μl; PLT = number of platelets ×10^3^/μl; WB, whole blood (pool of 250 ml); L-PRP, leukocyte platelet-rich plasma; L-PG, leukocyte platelet gel; L-PPP, leukocyte platelet-poor plasma (L-PPP); P-PRP, pure platelet-rich plasma; P-PG, pure platelet gel; P-PPP, pure platelet-poor plasma; FG, fibrin glue; PL, platelet lysate; T, thrombin-rich solution; nd, not detectable*.

### Bacterial Strains and Their Antibiotic Susceptibility Evaluation

Among the selected Gram-positive and Gram-negative microorganisms, different susceptibility profiles were observed and classified (Table 2) (70). One strain of *S. aureus* subspecies *aureus* and *S. cohnii* subspecies *cohnii*, one strain of *K. pneumoniae* subspecies *pneumoniae*, and one strain of *E. coli* resulted to be susceptible to the panel of antibiotics, and one strain of *S. aureus* subspecies *aureus, P. aeruginosa, K. pneumoniae* ssp. *pneumoniae*, and *E. coli* were MDR, while one strain of *S. aureus* subspecies *aureus, K. pneumoniae* ssp. *pneumoniae*, and *E. coli* resulted to be extensively drug-resistant. Only one strain of *P. aeruginosa* was PDR.

**Table 2:** Antimicrobial resistance profiles of microorganisms selected and identified by MALDI-TOF MS from canine wounds.

**Bacterial species**	**Resistance profiles**	**S**	**MDR**	**XDR**	**PDR**
*S. aureus* ssp. *aureus*	ENR-TE	x			
*S. cohnii* ssp. *cohnii*	AUG-P-OX	x			
*K. pneumoniae* ssp. *pneumoniae*	SXT-M	x			
*E. coli*	M	x			
*S. aureus* ssp. *aureus*	P-ENR-SXT		x		
*K. pneumoniae* ssp. *pneumoniae*	AUG-P-TE-M		x		
*E. coli*	SXT-TE-M		x		
*P. aeruginosa*	ENR-SXT-TE-M		x		
*S. aureus* ssp. *aureus*	AUG-OX-P-CDX-CFX- ENR-SXT-TE-S			x	
*K. pneumoniae* ssp. *pneumoniae*	AUG-P-CDX-CFX- ENR-SXT-TE-S-M			x	
*E. coli*	AUG-P-CDX-CFX-CEQ-ENR-SXT-TE-S-M			x	
*P. aeruginosa*	AUG-P-CDX-CFX-CEQ-ENR-CN-SXT-TE-AK-S-M-PB				x

*MALDI-TOF MS, matrix-assisted laser desorption ionization-time-of-flight mass spectrometry; S, susceptible; MDR, multidrug-resistant; XDR, extensively drug-resistant; PDR, pandrug-resistant; AUG 30 μg, amoxicillin and clavulanic acid; OX 1 μg, oxacillin; P 1 UI, penicillin G; CDX 30 μg, cefadroxil; CFX 30 μg, cefoxitin; CEQ 30 μg, cefquinome; ENR 5 μg, enrofloxacin; CN 10 μg, gentamicin; SXT 25 μg, sulfamethoxazole and trimethoprim; TE 30 μg, tetracycline; AK 30 μg, amikacin; S 300 μg, streptomycin; M 50 μg, metronidazole; PB 300 μg, polymyxin B; MIC VA, vancomycin; MIC TEC, teicoplanin*.

### Antibacterial Effect Evaluation of the Hemo-Components

#### Kirby–Bauer Method

**Figure 1:**
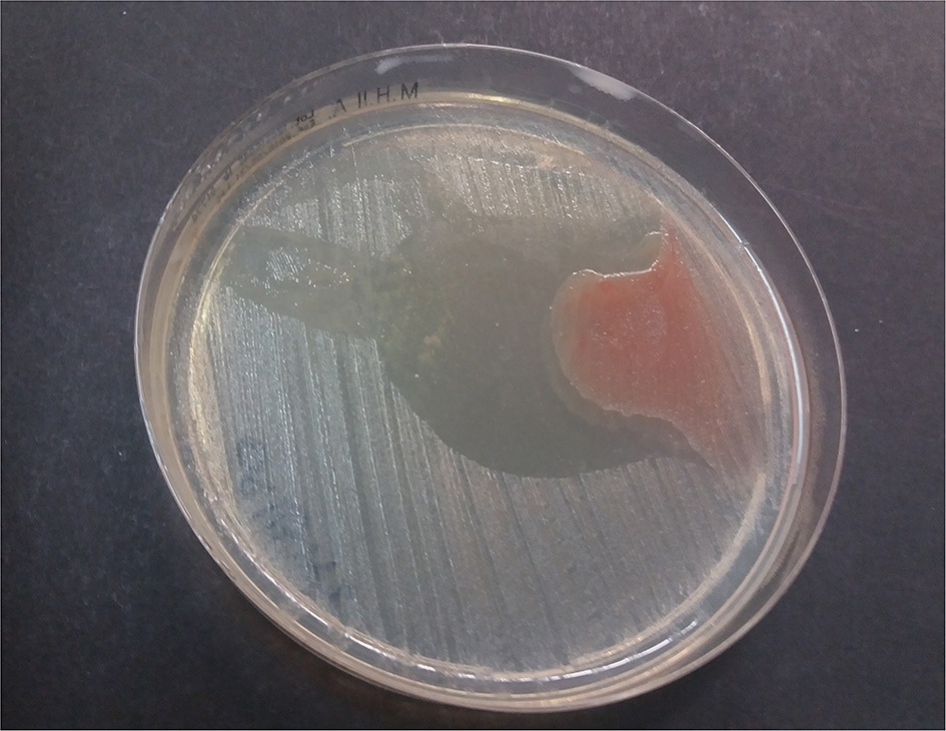
L-PG 500 μl effect against susceptible *K. pneumoniae* ssp. *pneumoniae* strain after 4 h at 37°C aerobically.

After 4 h of incubation at 37°C under aerobic conditions, no inhibition zones were observed for Gram-positive bacteria. Among Gram-negative microorganisms, some inhibition zones were observed for susceptible *K. pneumoniae* strains cultured with P-PG and L-PG. In particular, increased mean inhibition zones of 2.5 × 4.5 mm, 7.5 × 10 mm, and 8.5 × 50 mm were recorded for P-PG 35 μl, P-PG 500 μl, and L-PG 500 μl, respectively (Figure 1).

**Figure 2:**
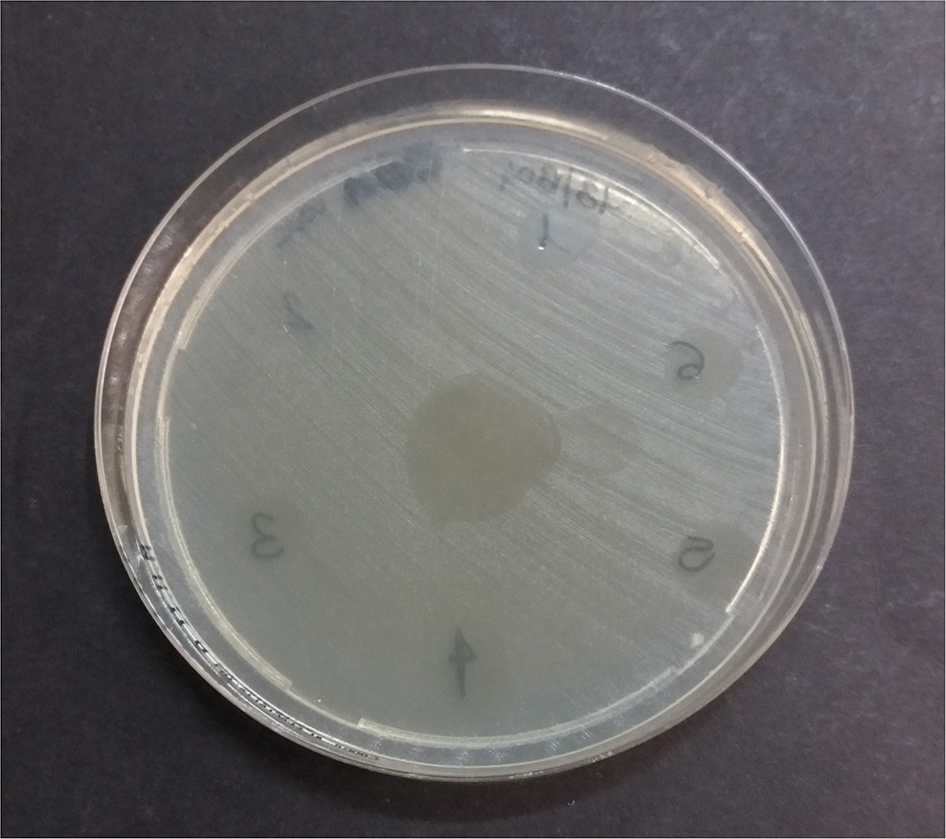
Multidrug-resistant *K. pneumoniae* ssp. *pneumoniae* inhibition zones by leukocyte-formulations after 4 h at 37°C aerobically. 1: platelet lysate; 2: thrombin; 3: L-PRP; 4: L-PRP plus thrombin; 5: L-PRP plus calcium gluconate; 6: L-PPP; in the center of the plate: L-PG.

**Figure 3:**
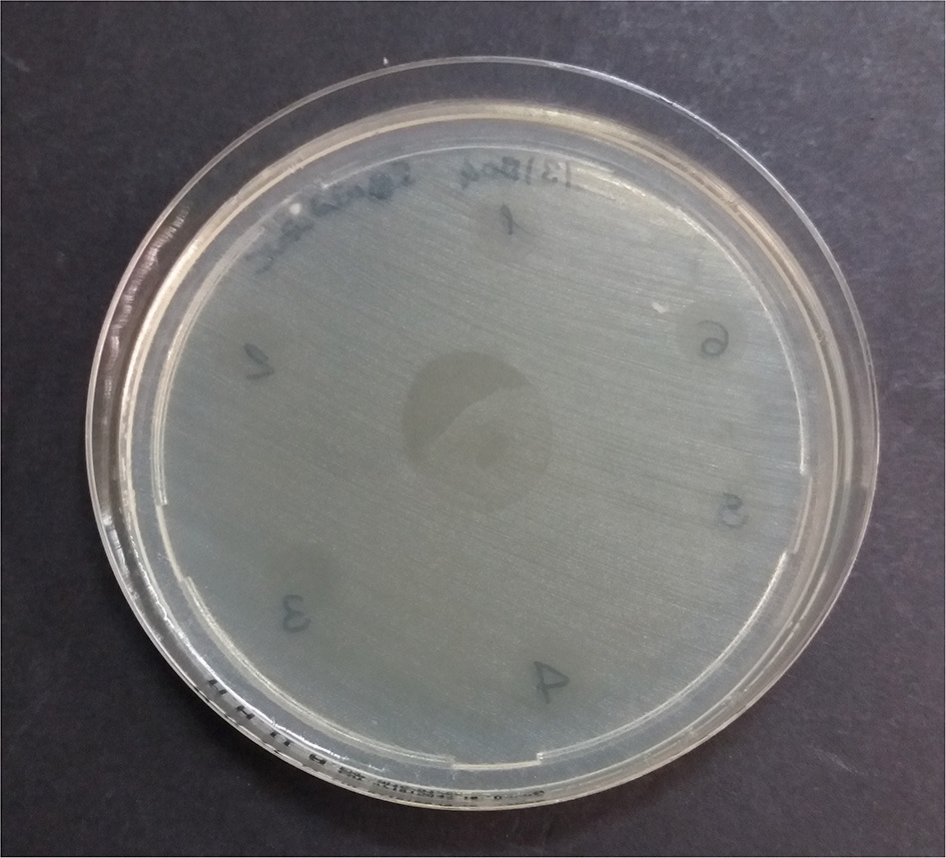
Multidrug-resistant *K. pneumoniae* ssp. *pneumoniae* inhibition zones by pure formulations after 4 h at 37°C aerobically. 1: fibrin glue; 2: calcium gluconate; 3: P-PRP; 4: P-PRP plus thrombin; 5: P-PRP plus calcium gluconate; 6: P-PPP; in the center of the plate: P-PG.

The MDR *K. pneumoniae* ssp. *pneumoniae* strain showed a zone of inhibition to all hemo-components, except for calcium gluconate, and P-PRP plus calcium and thrombin (Table 3, Figures 2, 3). PG showed a greater antibacterial effect, although non-significant differences were recorded between hemo-components both with and without leukocytes.

**Table 3:** Inhibition zones recorded by each hemo-component for multidrug-resistant *K. pneumoniae* ssp. *pneumoniae* after 4 h of incubation using the Kirby–Bauer method.

**Hemo-components**	**Inhibition zone (mm)**
Platelet lysate	9 × 10
Fibrin glue	9 × 12
L-PRP	8.5 × 9
P-PRP	9 × 9
L-PRP plus thrombin	9.5 × 9.5
P-PRP plus thrombin	9 × 9
L-PRP plus calcium gluconate	9 × 10
L-PPP	9.5 × 10
P-PPP	9 × 9
L-PG 35 μl	19 × 20
P-PG 35 μl	8 × 18
L-PG 500 μl	38 × 45
P-PG 500 μl	28 × 64

*L-PRP, leukocyte platelet-rich plasma; P-PRP, pure platelet-rich plasma; L-PPP, leukocyte platelet-poor plasma; P-PPP, pure platelet-poor plasma: L-PG, leukocyte platelet gel; P-PG: pure platelet gel*.

For extensively drug-resistant *K. pneumoniae* ssp. *pneumoniae* strains, inhibition zones of 2 × 3.5 mm, 4.5 × 37.5 mm, and 5 × 38 mm were observed for P-PG 35 μl, P-PG 500 μl, and L-PG 500 μl, respectively.

The L-PG 500 μl showed an antibacterial effect against the pan drug-resistant *P. aeruginosa* by recording an inhibition zone of 16.5 × 43 mm. Moreover, all the hemo-components gave areas of bacterial inhibition, except for thrombin and L-PRP with calcium gluconate against the extensively drug-resistant *E. coli* strain, while P-PPP and P-PG 35 μl, P-PG 500 μl, and L-PG 500 μl inhibited the growth of MDR and susceptible *E. coli*.

**Figure 4:**
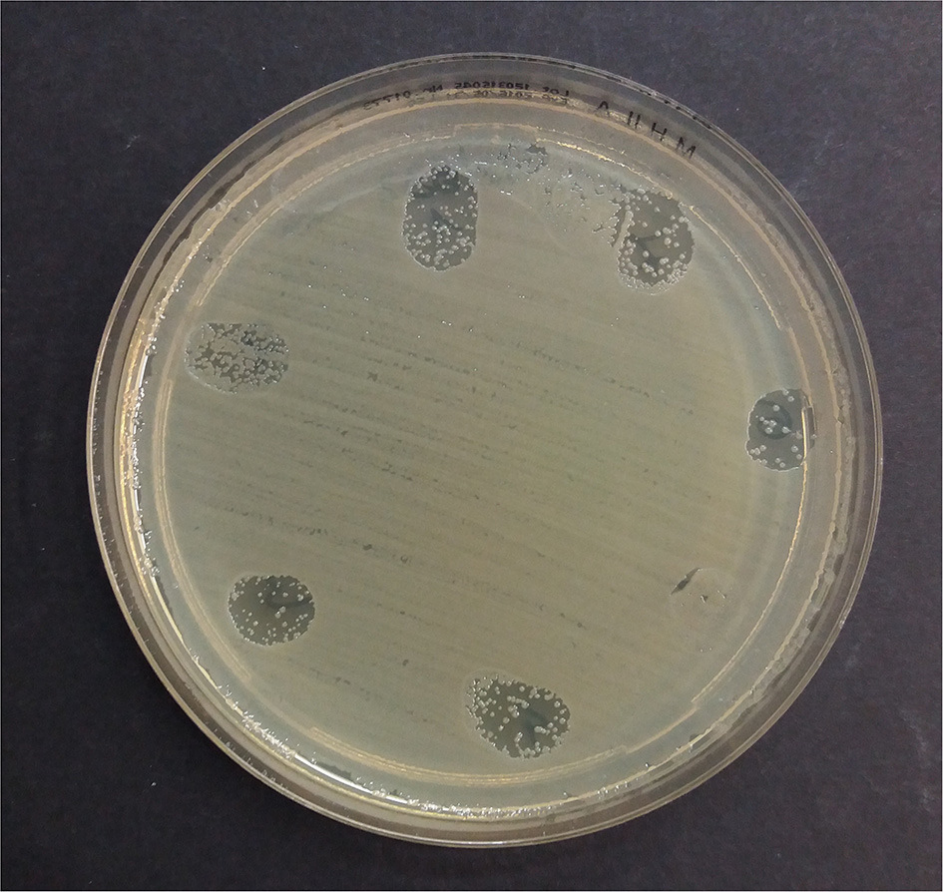
Leukocyte-formulations against the extensively resistant *E. coli* after 18 h at 37°C aerobically. 1: platelet lysate; 2: thrombin; 3: L-PRP; 4: L-PRP plus thrombin; 5: L-PRP plus calcium gluconate; 6: L-PPP; 7: L-PG.

The spectrophotometer reading after 18 h confirmed what was recorded after 4 h of incubation and revealed more defined inhibition zones. In particular, compared to the respective bacterial positive control, significantly reduced areas of bacterial growth (*p* < 0.05) were observed for the hemo-components against Gram-negative strains (Figure 4, Table 4).

**Table 4:** Inhibition zones (mm) recorded for Gram-negative bacteria after 18 h of incubation using the Kirby–Bauer method.

**Hemo-components**	**Inhibition zones (mm)**					
	***K. pneumoniae***		***E. coli***		***P. aeruginosa***	
	**MDR**	**XDR**	**MDR**	**XDR**	**MDR**	**PDR**
Platelet lysate	9 × 10		13 × 9		5 × 4	9 × 6
Fibrin glue				8 × 10		8 × 10
Thrombin					8 × 8	5 × 5
L-PRP		9 × 8	8.5 × 10.5		5.5 × 7	8.5 × 9.5
P-PRP		9 × 9			8 × 10	
L-PRP plus thrombin	9 × 9			9 × 12.5	6.5 × 6	7 × 7
P-PRP plus thrombin	9 × 9		8 × 10			
P-PRP plus calcium gluconate			8 × 10			
L-PRP plus calcium gluconate	8 × 9		10 × 11		4 × 4.5	6.5 × 7
L-PPP		9 × 9.5	8.5 × 10.5		5 × 7	8 × 9.5
P-PPP		9 × 9	8 × 10	6.5 × 8		
L-PG 35 μl	19 × 20	9 × 10		11 × 10	10.5 × 18	11 × 12
P-PG 35 μl	8 × 18					
L-PG 500 μl	38 × 45	19 × 20	18.5 × 10.5	16 × 10	20.5 × 20	21 × 22
P-PG 500 μl	28 × 64					

*L-PRP, leukocyte platelet-rich plasma; P-PRP, pure platelet-rich plasma; L-PPP, leukocyte platelet-poor plasma; P-PPP, pure platelet-poor plasma; L-PG, leukocyte platelet gel; P-PG, pure platelet gel*.

After 18 h of incubation, L-PG 35 μl, L-PG 500 μl, and P-PG 500 μl also showed a significant antibacterial effect (*p* < 0.05) against Gram-positive bacteria. Inhibition zones of 4 × 10 mm, 19 × 26 mm, and 50 × 22 mm were recorded for susceptible *S. aureus* ssp. *aureus* in the presence of L-PG 35 μl, L-PG 500 μl, and P-PG 500 μl, respectively. *S. cohnii* ssp. *cohnii* susceptible to the panel of antibiotics tested resulted to be inhibited by P-PG 500 μl (4 × 17.5 mm).

At 24 h, the size of each inhibition zones was confirmed.

#### Antibacterial Evaluation by Broth Inhibition by Microtiter Method

Reductions in bacterial growth (mean OD_540nm_ values) were also recorded with the microtiter method, as early as 4 h for Gram-negative microorganisms. This was confirmed by reading at 18 and 24 h. In relation to Gram affinity, different hemo-components induced a significant decrease in the growth of Gram-negative (0.897 vs. 1.604, t = 3.537 *p* = 0.001) and resistant bacteria (0.742 vs. 1.908, t = 4.506 *p* = 0.0005) compared with the corresponding bacterial growth in the absence of the hemo-component. At 18 h, thrombin significantly reduced the MDR *P. aeruginosa* growth (1.845 vs. 2.258, t = 41.094 *p* = 0.0001). The presence of leukocytes inhibited the bacterial growth more, but not statistically significant, compared with hemo-components without leukocytes. Significant differences were observed for MDR, XDR *K. pneumoniae, E. coli*, XDR, and PDR *P. aeruginosa* (Table 5).

**Table 5:** OD_540nm_ reductions in Gram-negative bacteria (mean OD_540nm_ bacterium plus hemo-component vs. mean OD_540nm_ bacterial control) recorded after 18 and 24 h of incubation by the broth microdilution method.

**Hemo-components**	**Time (h)**	**OD**_**540nm**_					
		***K. pneumoniae***		***E. coli***		***P. aeruginosa***	
		**MDR**	**XDR**	**MDR**	**XDR**	**MDR**	**PDR**
**Bacterial controls without hemo-components**	18	1.195	1.300	1.432	1.568	2.258	2.641
	24	1.413	1.440	1.512	1.338	2.340	2.653
Fibrin glue	18				1.366		
	24				0.932 ^*1^		
Thrombin	18					1.845 ^*2^	
	24					1.633 ^*3^	
L-PRP	18		1.290		1.498	2.002	2.230 ^*4^
	24				0.978 ^*5^		2.116 ^*6^
P-PRP	18		1.110			2.010	
	24		1.268			2.100	
L-PRP plus thrombin	18	1.123			1.448	2.200	2.640
	24	1.220					
P-PRP plus thrombin	18	1.104		1.398			
	24	1.300		1.400			
L-PRP plus calcium gluconate	18	0.901		1.400		2.188	2.500
	24	1.290		1.480		2.200	2.512
P-PRP plus calcium gluconate	18			1.369			
	24			1.408			
L-PPP	18		1.124	0.023 ^*7^			2.061 ^*8^
	24						2.019 ^*9^
P-PPP	18		1.180	0.035 ^*10^	1.014		2.260 ^*11^
	24						2.282 ^*12^
L-PG 10 μl	18						
P-PG 10 μl	24						
L-PG 20 μl	18	1.101^*13^					
P-PG 20 μl	24						
L-PG 40 μl	18	0.911	1.144	1.103 ^*14^		2.134	2.568
	24			1.104 ^*15^	0.970 ^*16^		
P-PG 40 μl	18	0.969			1.099 ^*17^		2.328 ^*18^
	24			1.176 ^*19^	1.236 ^*20^		
L-PG 180 μl	18	0.449 ^*21^	1.068 ^*22^	0.345 ^*23^	0.265 ^*24^	1.196 ^*25^	0.357 ^*26^
	24	0.480 ^*27^		0.350 ^*28^	0.272 ^*29^	1.926 ^*30^	1.029 ^*31^
P-PG 180 μl	18	0.505 ^*32^		0.085 ^*33^		1.12 ^*34^	0.643 ^*35^
		0.466 ^*36^	1.006 ^*37^	0.003 ^*38^	0.063 ^*39^	1.007 ^*40^	1.355 ^*41^

*At 18 h:*

*2 *(t = 41.094 p = 0.001)*;

*4 *(t = 30.398 p = 0.011)*;

*7 *(t = 23.895 p = 0.003)*;

*8 *(t = 27.730 p = 0.013)*;

*10 *(t = 7.001 p = 0.020)*;

*11 *(t = 27. 586 p = 0.001)*;

*13 *(t = 151.101 p < 10^−4^)*;

*14 *(t = 122.002 p = 0.0001)*;

*17 *(t = 22.985 p = 0.0023)*;

*18 *(t = 22.265 p = 0.002)*;

*21 *(t = 222.563 p < 10^−4^)*;

*22 *(t = 9.283 p = 0.011)*;

*23 *(t = 165.210 p < 10^−4^)*;

*24 *(t = 267.991 p < 10^−4^)*;

*25 *(t = 166.877 p < 10^−4^)*;

*26 *(t = 156.681 p < 10^−4^)*;

*32 *(t = 7.192 p = 0.019)*;

*33 *(t = 23.895 p = 0.002)*;

*34 *(t = 22.237 p = 0.002)*;

*35 *(t = 31.988 p = 0.001)*.

*At 24 h:*

*1 *(t = 34.203 p = 0.001)*;

*3 *(t = 175.258 p < 10^−4^)*;

5 *(t = 42.135 p = 0.001)*;

*6 *(t = 339.621 p < 10^−4^)*;

*9 *(t = 351.950 p < 10^−4^)*;

*12 *(t = 17.645 p = 0.003)*;

*15 *(t = 226.595 p < 10^−4^)*;

*16 *(t = 116.373 p = 0.0001)*;

*19 *(t = 186.653 p < 10^−4^)*;

*20 *(t = 168.233 p < 10^−4^)*;

*27 *(t = 452.334 p < 10^−4^)*;

*28 *(t = 734.907 p < 10^−4^)*

*29 *(t = 139.973 p = 0.0001)*;

*30 *(t = 48.647 p = 0.0004)*;

*31 *(t = 765.556 p < 10^−4^)*;

*36 *(t = 130.081 p = 0.0001)*;

*37 *(t = 142.865 p < 10^−4^)*;

*38 *(t = 65.491 p = 0.0002)*;

*39 *(t = 39.049 p = 0.001)*;

*40 *(t = 32.126 p = 0.001)*;

*41 *(t = 181.383 p < 10^−4^)*.

No significant bacterial reductions were observed when P-PRP, L-PRP, and P-PRP with thrombin, L-PRP and P-PRP with calcium gluconate, and L-PG 10 μl and P-PG 10 μl were applied for 18 and 24 h.

For Gram-positive bacteria, OD_540nm_ reductions were observed at 18 h for all *S. aureus* ssp. *aureus* strains in the presence of FG (1.025 vs. 1.439, t = 114.969 *p* = 0.0001) and 24 h (1.153 vs. 1.553, t = 222.159 *p* < 10^−4^) in comparison to the positive controls.

L-PG 10 μl reduced MDR *S. aureus* ssp. *aureus*, both at 18 h (1.15 vs. 1.481, t = 160.803 *p* < 10^−4^) and at 24 h (0.665 vs. 0.9765, t = 278.611 *p* < 10^−4^). L-PG 20 μl (0.622 vs. 0.955, t = 7.8347 *p* = 0.0159), L-PG 180 μl (−0.018 vs. 0.955, t = 46.344 *p* = 0.0005), and P-PG 180 μl (−0.4055 vs. 0.955, t = 11.783 *p* = 0.007) were able to reduce XDR *S. aureus* ssp. *aureus* growth. Similar significant reductions were confirmed for the P-PG 180 μl at 24 h (−0.354 vs. 0.976, t = 1.2 *p* < 10^−4^).

For hemo-components such as PL, FG, and thrombin, comparison of the mean ODs recorded at 18 and 24 h for all groups of bacteria showed a non-significant increase, except for FG for resistant bacteria, for which there was a reduction, although not significant.

**Figure 5:**
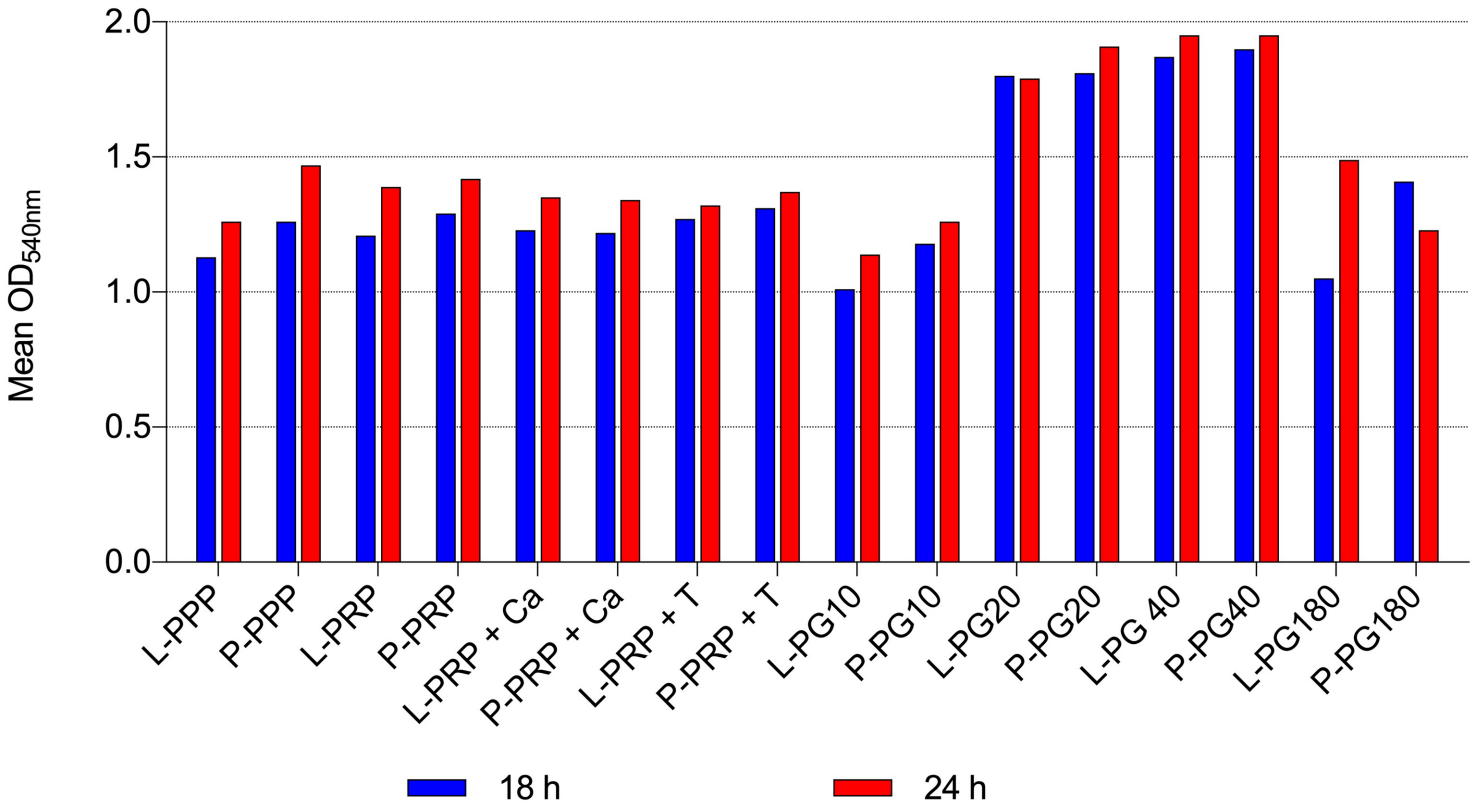
Mean OD_540nm_ values of Gram-positive bacteria observed after 18 and 24 h of incubation in presence of different non-transfusional hemo-components. L-PPP, leukocyte platelet-poor plasma; P-PPP, pure platelet-poor plasma; L-PRP, leukocyte platelet-rich plasma; P-PRP, pure platelet-rich plasma; L-PRP + Ca, leukocyte platelet-rich plasma with calcium gluconate; P-PRP + Ca, pure platelet-rich plasma with calcium gluconate; L-PRP + T, leukocyte platelet-rich plasma with thrombin-rich solution; P-PRP + T, pure platelet-rich plasma with thrombin-rich solution; L-PG10, leukocyte platelet gel 10 μl; P-PG10, pure platelet gel 10 μl; L-PG20, leukocyte platelet gel 20 μl; P-PG20, pure platelet gel 20 μl; L-PG40, leukocyte platelet gel 40 μl; P-PG40, pure platelet gel 40 μl; L-PG180, leukocyte platelet gel 180 μl; P-PG180, pure platelet gel 180 μl.

**Figure 6:**
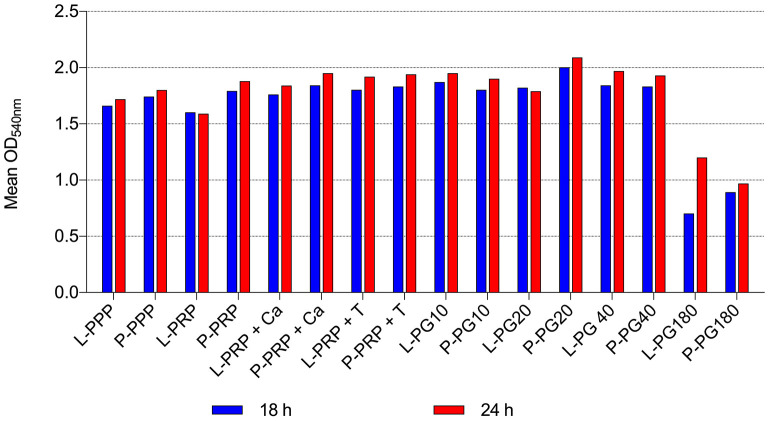
Mean OD_540nm_ values of Gram-negative bacteria observed after 18 and 24 h of incubation in the presence of different non-transfusional hemo-components. L-PPP, leukocyte platelet-poor plasma; P-PPP, pure platelet-poor plasma; L-PRP, leukocyte platelet-rich plasma; P-PRP, pure platelet-rich plasma; L-PRP + Ca, leukocyte platelet-rich plasma with calcium gluconate; P-PRP + Ca, pure platelet-rich plasma with calcium gluconate; L-PRP + T, leukocyte platelet-rich plasma with thrombin-rich solution; P-PRP + T, pure platelet-rich plasma with thrombin-rich solution; L-PG10, leukocyte platelet gel 10 μl; P-PG10, pure platelet gel 10 μl; L-PG20, leukocyte platelet gel 20 μl; P-PG20, pure platelet gel 20 μl; L-PG40, leukocyte platelet gel 40 μl; P-PG40, pure platelet gel 40 μl; L-PG180, leukocyte platelet gel 180 μl; P-PG180, pure platelet gel 180 μl.

**Figure 7:**
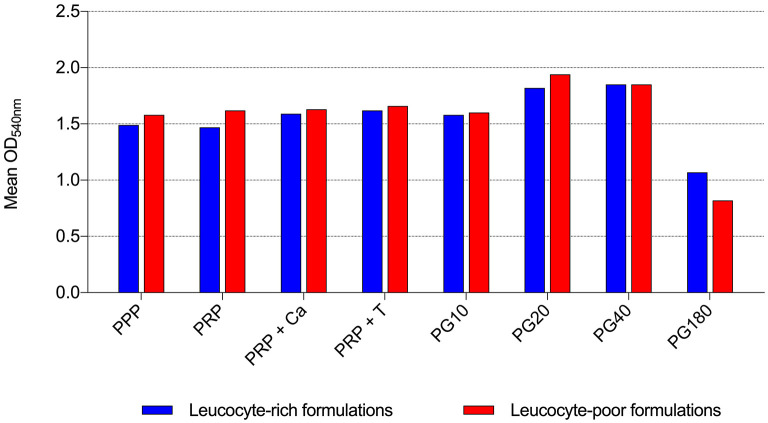
Mean OD_540nm_ values of leukocyte-rich formulations and leukocyte-poor formulations observed after 18 h of incubation in the presence of different non-transfusional hemo-components. PPP, platelet-poor plasma; PRP, platelet-rich plasma; PRP + Ca, platelet-rich plasma with calcium gluconate; PRP + T, platelet-rich plasma with thrombin-rich solution; PG10, platelet gel 10 μl; PG20, platelet gel 20 μl; PG40, platelet gel 40 μl; PG180, platelet gel 180 μl.

**Figure 8:**
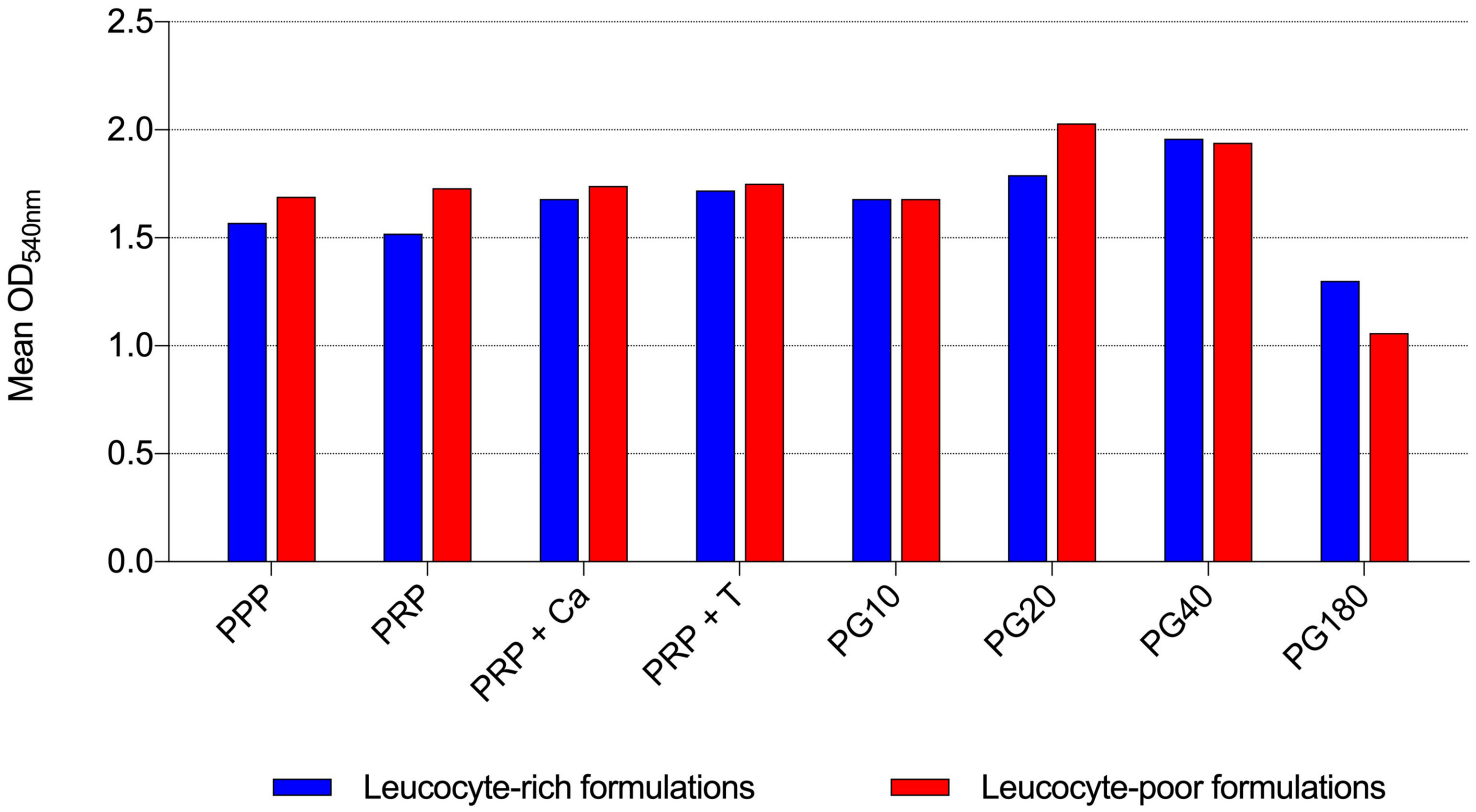
Mean OD_540nm_ values of leukocyte-rich formulations and leukocyte-poor formulations observed after 24 h of incubation in the presence of different non-transfusional hemo-components. PPP, platelet-poor plasma; PRP, platelet-rich plasma; PRP + Ca, platelet-rich plasma with calcium gluconate; PRP + T, platelet-rich plasma with thrombin-rich solution; PG10, platelet gel 10 μl; PG20, platelet gel 20 μl; PG40, platelet gel 40 μl; PG180, platelet gel 180 μl.

By the broth microdilution method, non-transfusional hemo-components with and without leukocytes showed similar bacterial properties toward all bacterial strains tested, although a greater but not significant reduction was observed for L-PG 180 μl, at both 18 h (*p* = 0.252) and 24 h (*p* = 0.306) (Figures 5–8).

## Discussion and Conclusions

Several studies have been conducted recently, mainly in human medicine, to demonstrate the antimicrobial effect of non-transfusional hemo-components for topical use.

Few studies have been performed *in vitro* regarding the potential antimicrobial effect of animal hemo-components (31, 51–53). One study showed antibacterial activity of thrombin-activated platelets in horses (51). Platelet-rich plasma from rabbits had strong *in vitro* antimicrobial properties against methicillin-susceptible and methicillin-resistant *Staphylococcus aureus*, Group A *Streptococcus*, and *Neisseria gonorrhoeae* (53). Moreover, Lopez evidenced a bacteriostatic effect of equine pure platelet-rich formulations (31). In addition, antibiofilm properties of equine platelet-rich plasma lysate were also highlighted (52).

Some *in vivo* experimental studies using animal models have also confirmed the potential antimicrobial effect of non-transfusional hemo-components (57, 61–63, 66). Platelet-rich plasma showed antimicrobial properties in rabbit models of osteomyelitis (61, 62) and postoperative spinal implant-associated infections (63). Platelet-rich plasma improved healing of experimentally infected surgical wounds in rats (57) and accelerated healing of canine skin wounds by exerting antibacterial activity, rapid reduction of inflammation, rapid re-epithelialization, and granulation tissue formation (66).

Based on this literature evidence on animals, this study assessed the antimicrobial activity of different hemo-components for non-transfusional use obtained from canine species against bacteria with different characteristics. To the authors' knowledge, this study represents the first steps conducted *in vitro* to evaluate the potential inhibitory effect of canine non-transfusional hemo-components on bacteria collected from canine wounds and classified according to the sensitivity/resistance spectrum toward a known panel of human and veterinary antibiotics.

Although this study was performed on a pool of WB from a limited number of dogs, the results will potentially contribute to enrich the knowledge on this topic, in order to provide some answers to important questions recently posed by different schools of thought.

The action of the hemo-components has been evaluated against Gram-positive bacteria (*S. aureus* ssp. *aureus, S. cohnii* ssp. *cohnii*) and Gram-negative (*P. aeruginosa, E. coli, K. pneumoniae* ssp. *pneumoniae*) isolated from biological samples of canine origin and previously classified as susceptible, resistant, or MDR to a panel of known antibiotics.

The potential antibacterial effect of the hemo-components has been evaluated by both agar gel diffusion (Kirby–Bauer), and broth inhibition using microtiter plates with spectrophotometer readings at a wavelength of 540 nm (OD_540nm_). PL, FG, and thrombin-rich solution were tested for the first time in veterinary medicine.

WB from dogs with degenerative joint disease who underwent intra-articular therapy with autologous PRP was used in this study; this choice was mainly determined by ethical reasons, but it also allowed us to be more adherent to daily clinical practice. To limit the impact of individual donor variations, as performed in other studies, a pool was created with WB collected from all dogs and all materials tested were obtained from the same pool of dogs (36, 48, 52).

In this study, the cell counts contributed to define the quality of hemo-components preparation procedures, supporting the hypothesis of some authors who relate the platelet concentration to the clinical regenerative effect as it is positively correlated with the concentration of growth factors (73–75). Conversely, an excessively high platelet concentration is considered counterproductive to the healing process, as it has the potential to inhibit the angiogenic process (76). The platelet fold increase from WB and the platelet concentration obtained in the final product could be considered rough measures to define the quality of the production process and of the PRP for clinical use, respectively (77).

Specifically, the platelet concentration in L-PRP was similar to what Mazzucco et al. considered a “reasonable compromise” for determining the quality of the PCs for regenerative medicine therapeutic purposes (77, 78).

Increases in platelet and leukocyte concentrations in PRP compared with baseline concentration in WB were similar to those obtained by Perego et al. (79).

Platelet and leukocyte contents in P-PRP were comparable to those in other studies (42).

Platelet and leukocyte concentrations were much lower (45-fold and 140-fold, respectively) in L-PPP than the L-PRP formulation, similarly to Burnouf who obtained a 50-fold decrease in leukocytes and a 100-fold decrease in platelets (45).

The potential antimicrobial role of leukocytes and platelets was evaluated by comparing the bacterial load (OD) inhibition values for PPP, PRP, PRP with calcium gluconate, PRP with thrombin, and PG in various amounts, in both leukocyte-rich formulations and leukocyte-poor (or pure) formulations. PPP, although rarely used in regenerative medicine for therapeutic purposes, has been tested to define the innate antimicrobial role of plasma or humoral immune response: the result of our study suggests that the antimicrobial bacteriostatic action, especially against Gram-negative bacteria, might be related to plasma components rather than the platelets. In this study, the presence of leukocytes has been shown not to be significant for the antimicrobial activity of hemo-components, although in the presence of leukocytes a reduction in growth was observed for a greater number of microorganisms than in the action of hemo-components without leukocytes. The effect of leukocytes in PCs is the focus of heated scientific debate. Many authors have suggested that the addition of white blood cells could enhance the antibacterial potential (47, 80–82). However, Anitua and collaborators have shown that the addition of leukocytes does not significantly improve the strong antibacterial properties. It is also possible that leukocytes could increase the inflammatory response at the site because they secrete metalloproteinases, pro-inflammatory proteases, and acid hydrolases (42). Other authors agree that the presence of leukocytes does not increase the antibacterial effect of PCs (31–33, 83). In our study, the presence of leukocytes is confirmed to be non-significant for the antimicrobial activity of non-transfusional hemo-components.

By the Kirby–Bauer method, the antibacterial effect of platelet gel (PG), both with and without leukocytes, has been documented as early as 4 h for some bacteria, confirmed at 18 h, especially for slow-growing bacteria, and remained constant until 24 h. Results obtained by the agar gel diffusion method (Kirby–Bauer) were confirmed by broth inhibition using microtiter plates and reading by spectrophotometry. Both L-PG and P-PG showed a reduction in the bacterial load of Gram-negative bacteria, also PDR *P. aeruginosa*.

Studies using the broth inhibition method did not agree with the observed results. Edelblute et al. showed no antibacterial activity of PG against *P. aeruginosa*, while significant bactericidal activity was observed against *S. aureus* and *Acinetobacter baumannii* (49). Drago et al. showed a lack of action of P-PRP, against *P. aeruginosa*; on the contrary, growth inhibition was recorded for Gram-positive and yeasts: *E. faecalis, Candida albicans, Streptococcus agalactiae*, and *Streptococcus oralis* (30).

Zones of inhibition were observed with L-PG, at the amount of 35 μl in both MDR Gram-negative microorganisms (*K. pneumoniae* ssp. *pneumoniae, P. aeruginosa*, and *E. coli*) and susceptible Gram-positive bacteria. In one study, 30 μl of L-PG induced, after 3 h and up to 48 h, a significant growth inhibition effect for *K. pneumoniae, P. aeruginosa, E. coli*, and *S. aureus* (45).

The results of our study are partly consistent with those described by Bielecki et al. who observed growth inhibition of *E. coli* and methicillin-susceptible and methicillin-resistant *S. aureus* strains with L-PG 12 μl. The measured inhibition zones were comparable to those of gentamicin and oxacillin, analog of methicillin (28). In contrast to our study, the same authors did not observe a reduction for *K. pneumoniae* and *P. aeruginosa* (28).

As the amount of PG tested increased, the inhibitory effect on bacteria also increased, because of the observed reduction in bacterial load. L-PG 500 μl decreased the growth of all microorganisms, except for methicillin-susceptible *S. aureus* ssp. *aureus* strain.

The potential antibacterial effect of leukocyte-poor or pure platelet gel (P-PG) was also tested in this study. At the amount of 35 μl, P-PG already showed an area of growth inhibition exclusively for multiresistant *K. pneumoniae* ssp. *pneumoniae*, whereas at the amount of 500 μl P-PG showed growth inhibition for more types of bacteria: this effect was observed for all bacterial strains except MDR *P. aeruginosa* and *S. conhii* ssp. *cohnii*.

Inhibitory action was also observed for formulations in the pre-activation phase (L-PRP, PRP-P), although not against Gram-positive bacteria.

In the inhibition zones, characterized by lower growth halos, a bacteriostatic action can be assumed, as for some antibiotic drugs.

A recent systematic review also observed a tendency of PCs to inhibit the growth of microorganisms during the first hours of incubation, whereas they did not seem to be able to completely break down the microbial load, indicating a bacteriostatic rather than a bactericidal activity (56).

On this aspect, in the presence of thrombin-activated PRP, Wu et al. observed a reduction in the number of *E. coli, P. aeruginosa*, and *K. pneumoniae* during the first 8 to 12 h, with the greatest reduction observed at 0 to 4 h. The number of bacteria increased again after the 4-h time point because the bacterial killing process was not complete; after this time point, bacterial growth exceeded the killing rate and growth continued until the stationary phase was reached at the 24-h time point (47).

It is interesting to note that PL, FG, and thrombin had an inhibitory action only against Gram-negative bacteria. This finding could open up innovative therapeutic possibilities, potentially exploiting combined therapies in a clinical scenario with infections sustained by these types of bacteria, sometimes rather aggressive and difficult to treat. Specifically, thrombin at the amount of 8 μl was able to create a visible zone of reduced growth for the two *P. aeruginosa* strains (MDR and PDR). The only strain of *S. aureus* ssp. *aureus* (susceptible to the panel of antibiotics) whose bacterial load was significantly decreased by FG was also moderately inhibited by PL and thrombin. Although studies on these hemo-components are very few in the literature, our findings are in contrast to Bielecki and collaborators who observed no inhibitory effect using 12 μl of thrombin against *S. aureus, E. coli, K. pneumoniae, P. aeruginosa*, and *E. faecalis* (28).

Other studies have shown that activation of platelets in PRP with thrombin leads to a significant increase in inhibitory activity on bacteria (51). In the same study, addition of thrombin to *E. coli* was shown not to affect bacterial growth, and addition of thrombin to PPP did not increase the inhibitory effect (51).

Although further studies will need to be done to clarify the mechanism of action, the thrombin-rich solution used in our study may have had antibacterial action by itself, because it is derived from autologous plasma, and it is not thrombin derived from complex chemical extraction.

Some studies have tested the potential link between the antibacterial effect and the platelet activation process with CaCl_2_, reaching discordant results (45, 48). According to Burnouf et al., activation of coagulation to prepare PG may reduce antimicrobial activity by consuming complement or other inhibitors or by releasing components that support bacterial proliferation (45). Contrariwise, Drago et al. observed that only activated materials were able to inhibit bacterial growth, suggesting that the activation of coagulation is a key step (48).

The antibacterial action against Gram-negative bacteria, observed for L-PPP, P-PPP, L-PRP, and P-PRP and the same with the addition of calcium gluconate and thrombin, is consistent with that documented by Burnouf et al. Using PPP, PRP, and PL, these authors obtained inhibitory action against *E. coli, P. aeruginosa*, and *K. pneumoniae*, but not against Gram-positive microorganisms, except for *S. aureus* (45). The zones of inhibition observed in our study showed a minimum area of 4 × 4.5 mm in contrast to the zones of inhibition of 0.5, 0.5–1, and >1 mm considered by Burnouf et al. (45). Tohidnezhad et al. also observed antibacterial action of PRP with thrombin for *E. coli* and *P. aeruginosa, Enterococcus faecalis, Bacillus megaterium*, and *Proteus mirabilis* (44). Li et al. found that PRP has strong antimicrobial properties *in vitro* against bacteria such as methicillin-susceptible and methicillin-resistant *Staphylococcus aureus*, Group A *Streptococcus*, and *Neisseria gonorrhoeae* (53).

The bacteriostatic action of the PPP, with and without leukocytes, was shown only for Gram-negative bacteria (*E. coli, P. aeruginosa*, and *K. pneumoniae* ssp. *pneumoniae*) belonging to the MDR and PDR groups. This finding suggests that the antimicrobial bacteriostatic action might be related to plasma components rather than platelets.

The mechanism of the antibacterial effect of PCs is not yet fully understood.

There are several studies suggesting that platelets play a very important role in the innate defense against the induction and progression of endovascular infections. This host defense capacity depends on the ability of platelets to release a group of antimicrobial peptides, known collectively as platelet protein microbicide, at the site of damage or endovascular infection. Indeed, platelets generate oxygen metabolites, including peroxide, hydrogen peroxide, and free radicals; are able to bind and aggregate microorganisms, improving the clearance of pathogens from the bloodstream; have chemotactic action on macrophages; participate in antibody-dependent cellular cytotoxicity functions against pathogens; and, finally, would release a series of potent antimicrobial peptides (84, 85).

In one study, PPP showed no impact on the growth parameters of any of the bacteria tested, whereas PRP showed an antibacterial effect with a strong correlation between platelet concentration and antibacterial activity (38). Mariani et al., instead, showed that both PRP and PPP inhibited bacterial growth for up to 2 h of incubation, but the effect of P-PRP was significantly higher than that of PPP (32).

One study proposed a possible molecular mechanism to explain the antimicrobial effect of PRP at least against *E. coli* and *P. mirabilis* through the detection of an antimicrobial peptide in PRP, the human beta-defensin 2 (hBD-2) (44). Another study showed that PCs induced a significant increase in hBD-2 expression in primary keratinocytes cell culture in a concentration- and time-dependent manner (86).

Several antibacterial peptides have also been identified in human platelets, including connective tissue-activating peptide 3 (CTAP-3), platelet factor 4 (PF-4), regulated upon activation–normal T-cell expressed and secreted protein (RANTES), thymosin β-4 (Tβ-4), platelet basic protein (PBP), fibrinopeptide A (FP-A), and fibrinopeptide B (FP-B) (87–89).

Drago et al. also tested P-PPP against Gram-positive bacteria isolated from the oral cavity obtaining similar, although slightly lower, results to P-PRP, whereas platelets alone showed no antibacterial activity (48). The authors concluded that the antimicrobial activity of PCs against *E. faecalis, S. agalactiae, S. oralis*, and *S. aureus* is supported by a synergy of plasma components and platelet-derived factors (48).

In our study, in relation to the different groups of microorganisms, significant reductions were observed only for Gram-negative MDR bacteria in the presence of PG 180 μl, both with and without leukocytes. For P-PG 180 μl, the inhibitory action was also documented for Gram-negative PDR bacteria.

The results of this study are certainly very encouraging and stimulate further studies to understand the mechanism of antibacterial effect. A greater knowledge of the mechanism of action could bring considerable advantages for potential practical applications, being able to better outline the indications, the limits, and the conditions of practical use, also for the antibacterial effect in addition to the consolidated regenerative effect of the non-transfusional hemo-components.

To justify the antibacterial effect of hemo-components, future research can be ideally oriented to the investigation of potential plasma factors, ideally starting from the peptides already identified for the human species but considering that there could be differences between the human and canine species, as evidenced by this study regarding the antibacterial effect.

Our study confirmed the hypothesized antibacterial properties of canine non-transfusional hemo-components. The bacteriostatic effect appeared to be higher against Gram-negative bacteria. The presence of neither leukocytes nor platelets seems to be essential for the antibacterial effect. Although the interaction of hemo-components with microbial pathogens needs further investigations because the exact mechanism responsible for the antibacterial activity is not yet fully known, non-transfusional hemo-components have been shown to represent a useful natural substance for infection control, especially at surgical sites in the immediate postoperative period. The emergence of multidrug-, extensively drug-, and pan drug-resistant bacteria poses a significant health and economic threat for animals and humans. Therefore, local application of non-transfusional hemo-components could support the action of molecules with antibiotic activity and represent a suitable alternative to control MDR pathogens.

## Data Availability Statement

The raw data supporting the conclusions of this article will be made available by the authors, without undue reservation.

## Ethics Statement

The animal study was reviewed and approved by Animal Welfare Body of the University of Camerino. Written informed consent was obtained from the owners for the participation of their animals in this study.

## Author Contributions

A-RA and AT contributed to the conception and design of the study. CI organized the database. ES and AT recruited the animals and prepared the hemo-components. A-RA, CI, AC, and ML performed the bacteriological evaluations. A-RA performed the statistical analysis. A-RA, CI, and AT wrote the first draft of the manuscript. AC, CR, GM, GR, LG, and VC wrote sections of the manuscript. All authors contributed to manuscript revision, read, and approved the submitted version.

## Funding

This work was supported by a research grant from the University of Camerino, Italy (Fondo di Ateneo per la Ricerca 2014.2015).

## Conflict of Interest

The authors declare that the research was conducted in the absence of any commercial or financial relationships that could be construed as a potential conflict of interest.

## Publisher's Note

All claims expressed in this article are solely those of the authors and do not necessarily represent those of their affiliated organizations, or those of the publisher, the editors and the reviewers. Any product that may be evaluated in this article, or claim that may be made by its manufacturer, is not guaranteed or endorsed by the publisher.
